# Epidemiology and Characteristics of *Rickettsia australis* (Queensland Tick Typhus) Infection in Hospitalized Patients in North Brisbane, Australia

**DOI:** 10.3390/tropicalmed2020010

**Published:** 2017-04-15

**Authors:** Adam Stewart, Mark Armstrong, Stephen Graves, Krispin Hajkowicz

**Affiliations:** 1 Department of Infectious Diseases, Royal Brisbane and Women’s Hospital, Butterfield St & Bowen Bridge Rd, Herston, Herston 4029, Australia; mark.armstrong@health.qld.gov.au (M.A.); krispin.hajkowicz@health.qld.gov.au (K.H.); 2 Australian Rickettsial Reference Laboratory, Barwon Health, Geelong Hospital, Bellerine Street, PO BOX 281, Geelong, Victoria 3220, Australia; graves.rickettsia@gmail.com; 3 School of Medicine, University of Queensland, St Lucia, Queensland 4072, Australia

**Keywords:** tick-borne diseases, *Rickettsia* infections, epidemiology, Queensland, Australia

## Abstract

Queensland tick typhus (QTT; *Rickettsia australis*) is a spotted fever group (SFG) rickettsial infection endemic to Australia. It is an underreported and often unrecognized illness with poorly-defined epidemiology. This article describes epidemiological features and the geographical distribution of QTT in hospitalized patients. Cases of QTT were identified retrospectively from 2000–2015 at five sites in Northern Brisbane through a pathology database. Included cases had a four-fold rise in SFG-specific serology, a single SFG-specific serology ≥256 or an SFG-specific serology ≥128 with a clinically consistent illness. Of the fifty cases identified by serology, 36 were included. Age ranged from 3–72 years (with a mean of 39.5 years) with a male-to-female ratio of 1:1.1. Fifteen of 36 (42%) study participants had hobbies and/or occupations linked with the acquisition of the disease. Seventeen of 36 (47%) identified a tick bite in the days preceding presentation to hospital, and reported exposure to a known animal host was minimal (25%). QTT infection occurred throughout the year, with half of the cases reported between April and July. Recent ecological and sociocultural changes have redefined the epidemiology of this zoonotic illness, with areas of higher infection risk identified. Heightened public health awareness is required to monitor QTT disease activity.

## 1. Introduction

Queensland tick typhus (QTT) is an important cause of febrile illness; particularly among those exposed to the eastern coastal bushlands in Australia [1]. Currently, a diagnosis of any rickettsial infection does not require compulsory reporting in Australia, making disease surveillance incredibly difficult and inaccurate [2,3]. Although its true epidemiological impact is unknown, recently identified socio-ecological drivers of rickettsial disease could re-define QTT as a public health threat [1]. In addition, recent serological surveys point to a disease burden along the eastern coast that is greater than previously realized [1,4]. The geographical distribution and boundaries of QTT infection continue to be pushed further along the coastline, as well as inland [5]. As our understanding of host-vector interactions and ecological factors that drive disease emergence improves, informed public health interventions will become pivotal.

## 2. Materials and Methods

Study patients were both adults and children identified retrospectively during the period of January 2000 to January 2015 at any of the five sites in Metropolitan North Hospital and Health Service (MNHSS) in Queensland, Australia via the local pathology database. Medical records were reviewed for each identified case. Epidemiological and clinical data were recorded onto a standardized electronic case report form. Clinical features were considered present only if their presence was documented in the medical record. Confirmed or probable cases of QTT included those with a greater or equal to a four-fold rise in spotted fever group (SFG)-specific serology; a single SFG-specific serology ≥256; or an SFG-specific serology ≥128 with a clinically consistent illness [5]. A clinically consistent illness was defined as a presentation typical for QTT, without a more likely diagnosis being apparent, as well as a good clinical response to doxycycline or another appropriate antibiotic. Cases were further excluded if serology results represented an old infection, an acute illness thought to be acquired overseas, or a known cause of antibody cross-reactivity to *Rickettsia*-specific antigens (e.g. autoimmune conditions). The indirect microimmunofluorescence assay (IFA) utilizing an SFG-specific *Rickettsia rickettsii* antigen was used in serological testing.

The study was conducted in accordance with the Declaration of Helsinki, and the protocol was approved by the Royal Brisbane & Women’s Hospital Ethics Committee (HREC/14/QRBW/432).

## 3. Results

Of the 50 cases identified by serological criteria alone through the pathology database, 36 were included after the exclusion criteria were applied. Two patient’s medical records were destroyed and thus inaccessible.

### 3.1. Demographics

Of the studied patients, age ranged from 3–72 years (with a mean of 39.5 years) and sex distribution was approximately equal between males and females with a male-to-female ratio of 1:1.1. Fifteen of 36 (42%) study participants had hobbies and/or occupations that were linked with the acquisition of the disease, including gardening, bushwalking/orienteering, camping, and working as a botanist, wildlife ranger, groundsman, or farm worker/grazier. Eleven of 36 (31%) patients acquired their infection in association with travel away from their primary residence. Four of 36 (11%) were noted to live on a property with dense surrounding bushland. Moreover, 17 out of 36 (47%) reported a tick bite in the days preceding presentation to hospital, and 3 out of 36 (8%) reported multiple tick bites in the past, usually associated with their occupation or place of residence. Reported exposure to vertebrate animals on history was only 9 out of 36 (25%), which included exposure to cattle, dogs and horses (Table 1). Of note, eight patients reported no known risk factors for acute rickettsial infection.

### 3.2. Distribution of Disease

**Figure 1:**
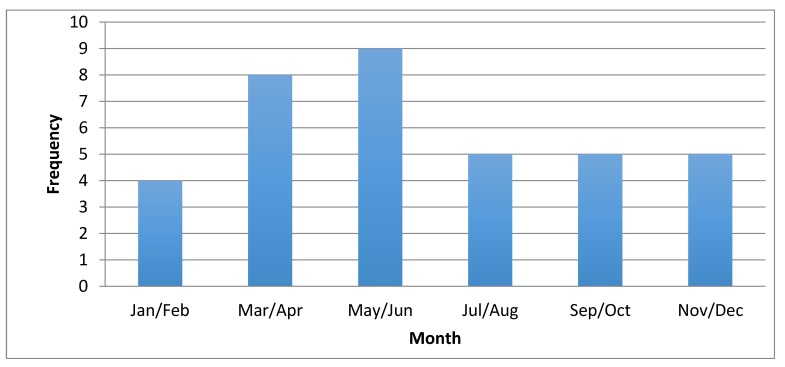
Seasonal variation in QTT infection in 36 hospitalized patients with Queensland tick typhus (QTT).

**Figure 2:**
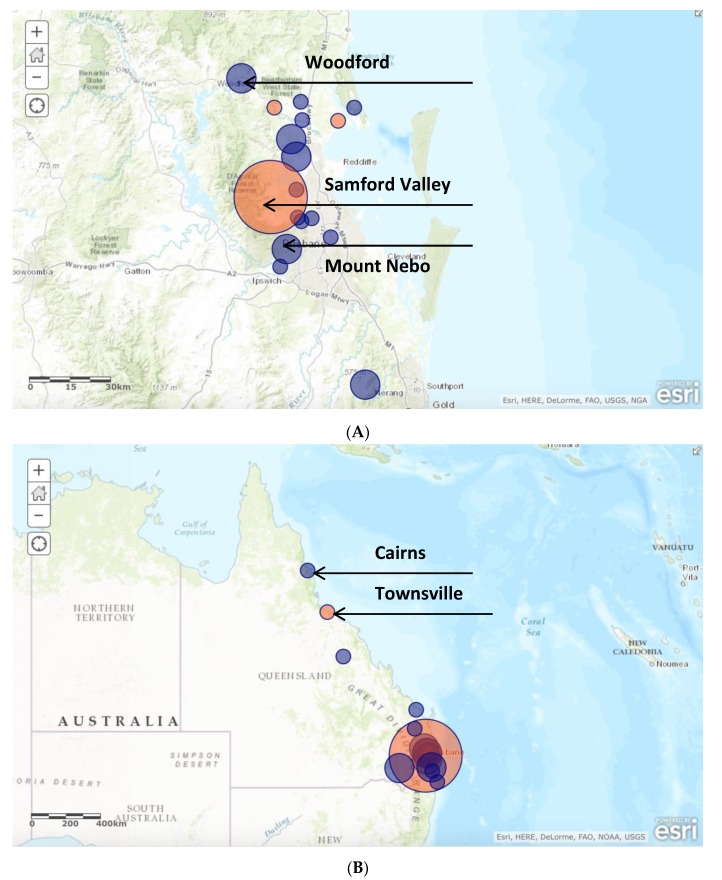
Location of the 36 patients who presented to the five study sites with acute *Rickettsia australis* infection within southeast (**A**) and greater Queensland (**B**) Note: Size of circle indicates relative frequency of infection; orange circles indicate where cases of severe infection occurred.

Eighteen of 36 (50%) cases occurred between April and July; 10/36 (28%) occurred from September to December; and 8/36 (22%) occurred between January and March (Figure 1). To determine the distribution of disease, the postcode of the patient’s primary residence was recorded, unless there was a clear history of preceding travel to another place where the tick bite had occurred; in this case, the postcode from the site where the infection was acquired was entered into the database. A high density of infection occurred in the Samford Valley, Woodford and Mount Nebo areas (Figure 2). Eleven of 36 (31%) infections were acquired in this area alone. Six of 36 (8%) were located in the Narangba and Morayfield areas, 5–10 kilometers from the coastal area of Samford. Other infections were sporadically distributed along the eastern coast of Queensland and northern New South Wales. Interestingly, of those requiring intensive care unit (ICU) admission (4/36), 75% lived or acquired their infection within a five-kilometer radius of each other, and presented during the March/April period.

## 4. Discussion

Defining the distribution of each SFG rickettsial disease in Australia has been a difficult task [1]. This is largely due to inadequate reporting of infection, as well as low recognition and testing in the community and hospital settings [3,6]. In addition, there may be a larger pool of subclinical infection [1]. We relied on sporadic independent serological surveys to guide our current understanding; these are few and far between [3]. Even these can be unreliable due to assay cross-reactivity with other pathogenic and non-pathogenic rickettsia, as well as with bacteria (e.g., *Proteus species*) [7]. This report is among the few current studies documenting the features of *R. australis* infection; however, it carries significant limitations due to its retrospective nature. 

Although *R. australis* infection can occur throughout the year, in this study half of the cases of QTT occurred between April and July, during the seasons of autumn and winter in Australia. This finding appeared to contradict previous reports of increasing incidence of QTT during the summer and spring months [8]. Adult female *Ixodes* spp. ticks (vector for *R. australis*) are most abundant in Queensland from October to December [9]. The reasons for this are unclear, although factors influencing vector distribution and behavior such as rainfall and climate change may play a role. 

When mapping out the geographical distribution of QTT among the 36 identified cases, the postcode of the site where the infection or tick bite was thought to have occurred was recorded. If this was not immediately apparent on chart review, the patient’s place of residence was used as a surrogate. The major limitation to this approach was that the tick bite was often not acquired from home, and often results from occupational or recreational exposures were not always elicited on history. Nonetheless, infection occurred along eastern coastal Queensland and northern New South Wales with none reported more than 20 kilometers inland—a finding that is consistent with previous reports [2]. A focal hyperendemic area of QTT infection was identified spanning the Samford Valley, Woodford and Mount Nebo regions, accounting for 31% of cases. A further 8% of cases were located within a 5–10 kilometer distance from this area. This identifies an area where ecological conditions for *Ixodes holocyclus* and mammalian hosts are optimal, causing increased infection among susceptible humans [1]. Increasing urbanization of this area over the last few decades has been a major contributor to this phenomenon [1]. It is likely that this will be seen in multiple locations along coastal Australia as population densities change and encroach into new areas. 

The mean age of infection was 39.5 years (median 36.1 years), although there was a wide range observed (3–72 years). Extremes of age did not appear to increase the risk of infection. The male-to-female ratio was 1:1.1, which are in contrast to previous reports that revealed a male predominance (2:1) [8]. This could represent a change in occupation and recreational activities among women over the past half century, which is known to highly influence the risk of infection. Given the large number of mammalian host species for *Ixodes* spp. ticks and their predilection for wet forested areas in certain times of the year, certain human activities are high risk for acquiring infection [8,10]. Nearly half of the study participants acquired their infection through occupational or recreational activities, which was consistent with previous studies [8]. No new high-risk human behaviors were identified. 

Tick bite is still a useful guide for identifying those with possible QTT, with nearly half reporting this in their history. Exposure to vertebrate animal hosts may be of less utility in diagnosis; although, this aspect of the patient’s history was frequently missed or poorly documented. Multiple tick bites were reported by a minority of patients, especially those living in tick-dense areas, and may be particularly important for identifying those at risk of severe disease and sepsis [1]. Many patients discharged from hospital were given advice to avoid ticks, although none had documented lifestyle changes on follow-up; moreover, no reinfections occurred. It is difficult to conclude any benefit from this advice.

## 5. Conclusions

*Rickettsia australis* and QTT is an evolving disease with a growing public health importance [1]. A lack of current epidemiological data on its incidence and distribution limits our understanding of the disease and options for beneficial public health interventions. This study has demonstrated the current epidemiology of this infection and has identified a new area of intense QTT endemicity in eastern coastal Queensland. SFG rickettsial infections should be notifiable diseases in Australia. Furthermore, prospective studies refining our understanding of the risk of infection and hospitalization of QTT need to be carried out. In addition, serological surveys of both animal hosts and humans would identify potential hot-spot areas of QTT to limit future disease. 

## Figures and Tables

**Table 1:** Demographic and historical features of 36 patients with Queensland tick typhus (QTT).

Characteristic	Value
Age	3–72 years (Mean 39.5, Median 36.1)
Sex	Male-to-female ratio 1:1.1
- Male	17/36 (47%)
- Female	19/36 (53%)
Occupation	
- Groundsman/ranger	3/36 (8%)
- Farming	3/36 (8%)
Hobby/activity	
- Camping	3/36 (8%)
- Gardening	3/36 (8%)
- Bushwalking	2/36 (6%)
- Other	1/36 (3%)
Residence on acreage/property	4/36 (11%)
Recent travel (e.g., holiday)	11/36 (31%)
Tick bite	17/36 (47%)
- Single	14/36 (39%)
- Multiple	3/36 (8%)
Frequency of known risk factors	
- 0	8/36 (22%)
- 1	13/36 (36%)
- 2	13/36 (36%)
- 3	3/36 (8%)
